# Implants in patients with oral manifestations of autoimmune or muco-cutaneous diseases – A systematic review

**DOI:** 10.4317/medoral.22786

**Published:** 2019-03

**Authors:** Frank P. Strietzel, Andrea M. Schmidt-Westhausen, Konrad Neumann, Peter A. Reichart, Jochen Jackowski

**Affiliations:** 1 Charité – Universitaetsmedizin Berlin / Charité Centre 3 for Dental, Oral, and Maxillary Medicine, Department for Oral Medicine, Dental Radiology, and Oral Surgery; 2 Charité – Universitaetsmedizin Berlin, corporate member of Freie Universitaet Berlin, Humboldt-Universitaet zu Berlin, and Berlin Institute of Health (BIH), Institute of Biometry and Clinical Epidemiology; 3 Department of Oral Surgery and Dental Emergency Care, Faculty of Health, School of Dentistry Witten/Herdecke University, North Rhine-Westphalia

## Abstract

**Background:**

To give an overview on implant survival rates in patients with oral manifestations of systemic autoimmune (oral Lichen planus (oLp), Pemphigus (Pe)), muco-cutaneous (Epidermolysis bullosa (EB)), autoimmune multisystemic rheumatic diseases (Sjögren´s syndrome (SjS), systemic Lupus erythematosus (sLE), or systemic Sclerosis (sSc)).

**Material and Methods:**

Systematic literature review (PubMed/Medline, Embase) using MESH and search term combinations, published between 1980 and August 2018 in English language reporting on dental implant-prosthetic rehabilitation of patients with oLp, Pe, EB, SjS, sLE, sSc, study design, age, gender, follow-up period (≥ 12 months), implant survival rate. Implant-related weighed mean values of implant survival rate (wmSR) were calculated.

**Results:**

After a mean follow-up period (mfp) of 44.6 months, a wmSR of 98.3 % was calculated from data published for patients with oLp (100 patients with 302 implants). Data of 27 patients (152 implants) with EB revealed wmSR of 98.7 % following mfp of 32.6 months. For 71 patients (272 implants) with SjS, wmSR was 94.2 % following a mfp of 45.2 months, and for 6 patients (44 implants) with sSc, wmSR was 97.7 % after mfp of 37.5 months. One case report on one patient each with Pe (two implants) as well as sLE (6 implants) showed 100 % SR following at least 24 months.

**Conclusions:**

Guidelines regarding implant treatment of patients with oLp, Pe, EB, SjS, sLE or sSc do not exist nor are contraindicating conditions defined. Implant survival rates of patients affected are comparable to those of healthy patients. For implant-prosthetic rehabilitation of patients with Pe and sLE no conclusions can be drawn due to lack of sufficient clinical data. Implant-prosthetic treatment guidelines regarding healthy patients should be strictly followed, but frequent recall is recommended in patients affected with oLp, SjS, EB, SSc, Pe or sLE.

** Key words:**Dental implants, implant supported prosthesis, oral lichen planus, Sjögren´s syndrome, epidermolysis bullosa, systemic sclerosis, pemphigus, systemic lupus erythematosus.

## Introduction

Survival and success rates of implant-prosthodontic rehabilitations in patients without compromised general health conditions have been reported remarkably high even in mid- and long-term observations, after follow-up periods of up to 10 or exceeding 10 years (1).

Although several conditions still are considered risk factors for dental implant prognosis in medically compromised patients, there are only few absolute contraindications for this treatment option. Thus, disease control of conditions increasing the risk, and individualized risk-benefit assessment prior to dental implant therapy may be considered more important than the disorder or risky condition itself (2). However, dental implant therapy is a viable treatment option even in these patients, revealing satisfying success rates of implant-borne prosthodontic treatment, suggesting that these patients might have a benefit from this treatment option. Moreover, these results encourage to overcome limitations of indications for dental implant treatment under reasonable and careful stepwise treatment regimen as there are interdisciplinary approach, full therapy adherence by the patients as well as strict conditions of tight recall intervals.

Oral manifestations of certain non-infectious systemic diseases – among them autoimmune, ulcerative, bullous, or fibrous – are frequently aggravated by secondary effects of the underlying pathosis in patients suffering from oral Lichen planus (oLp), pemphigus (Pe), Epidermolysis bullosa (EB), Sjögren´s syndrome (SjS), systemic Lupus erythematosus (sLE), or systemic sclerosis (sSc).

These patients suffer from inflammatory, ulcerous, erosive lesions, sicca symptoms, scar formation, or limitation of mouth opening. Secondary effects of these diseases are compromised eating and swallowing, xerostomia, enhanced risk of caries and periodontitis decay, alveolar ridge atrophy, scar tissue formation, enhanced risk of oral squamous cell carcinoma, and comprise side effects of systemic anti-inflammatory or immune-modulating medication. Thus, all these patients have in common a significant reduction of quality of life. Moreover, oral hygiene, dental treatment interventions and especially wearing mucosal-borne dentures are impeded. Therefore, the question arises, if these patients might benefit from implant-borne prostheses, to avoid extensive contact between prostheses and the oral mucosa.

The aim of this systematic review of the literature was to reveal, if implant prosthetic treatment of patients suffering from oLp, Pe, EB, SjS, sLE or sSc can be considered a promising treatment option.

## Material and Methods

The focused question was: which implant survival rates were reported in patients suffering from mucocutaneous or autoinflammatory diseases oLp, Pe, EB, SjS, sLE or sSc? 

A systematic literature search was performed, using electronic literature databases (PubMed/Medline, Embase) and MESH and search term combinations (see  Table 1). The systematic review of the literature was done related to the PICO format. Publications reporting on patients suffering from oLp, Pe, EB, SjS, sLE or sSc (population), undergoing clinical use of dental implant-prosthetic rehabilitation (intervention) and revealing study design, age, gender, number of patients and implants, follow-up period of or exceeding 12 months and implant survival rate (outcome), and published between 1980 and December 2017 in English or German language were included. A control group was lacking in most of publications. Publications were excluded, if formation of malignant tumour in the head and neck region interfered with implant or patient survival.

**Table 1:**
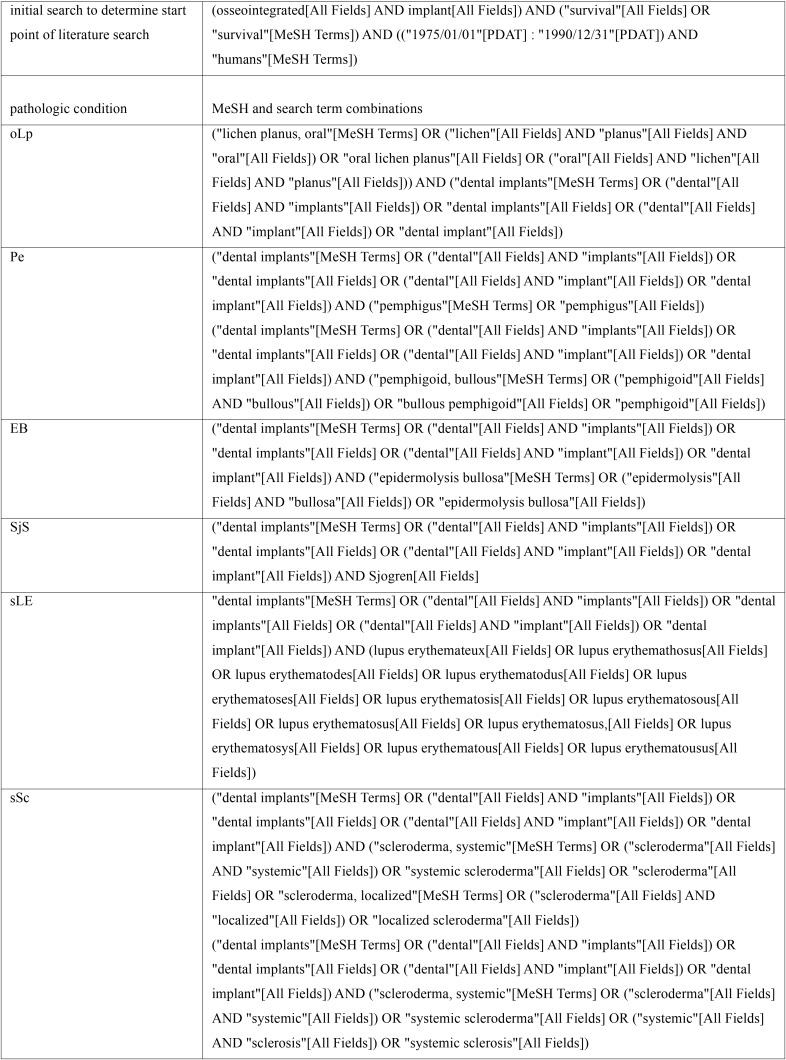
MeSH and search term combinations of literature search utilizing electronic databases.

Publications were identified and abstracts were evaluated independently by two reviewers (FPS and AMSW) by formal consideration of inclusion criteria and due to content matching the search purposes. Agreement of inclusion or exclusion of publications by two reviewers was expressed by calculation of the κ-value. After reaching full consent between the reviewers regarding the screening of abstracts, publications were selected for inclusion into further analysis.

Data reporting on implant survival, patients and implants numbers and duration of observational periods were retrieved from included publications. Weighed mean values of age (patient-related) and of implant-related implant survival rate (wmSR) and observational periods (mOP) were calculated.

For meta-analysis funnel and forest plots for all publications and for the subgroups of publications with data regarding oLp, SjS, EB, and sSc were created. The funnel plot revealed that there is no evidence for publication bias if four publications were excluded from meta-analysis. Forest plots showed 95% confidence intervals for the pooled implant failure rate per year and for the failure rate of each study. Moreover, I2 (measure for heterogeneity) was calculated for each meta-analysis.

The statistical analysis was carried out using SPSS 22.0, R version 3.4.3 and the R package “meta”.

 Table 2,  Table 2 continue,  Table 2 continue-1 contains the PRISMA checklist regarding the methodology of the systematic review process and data analyses.

**Table 2:**
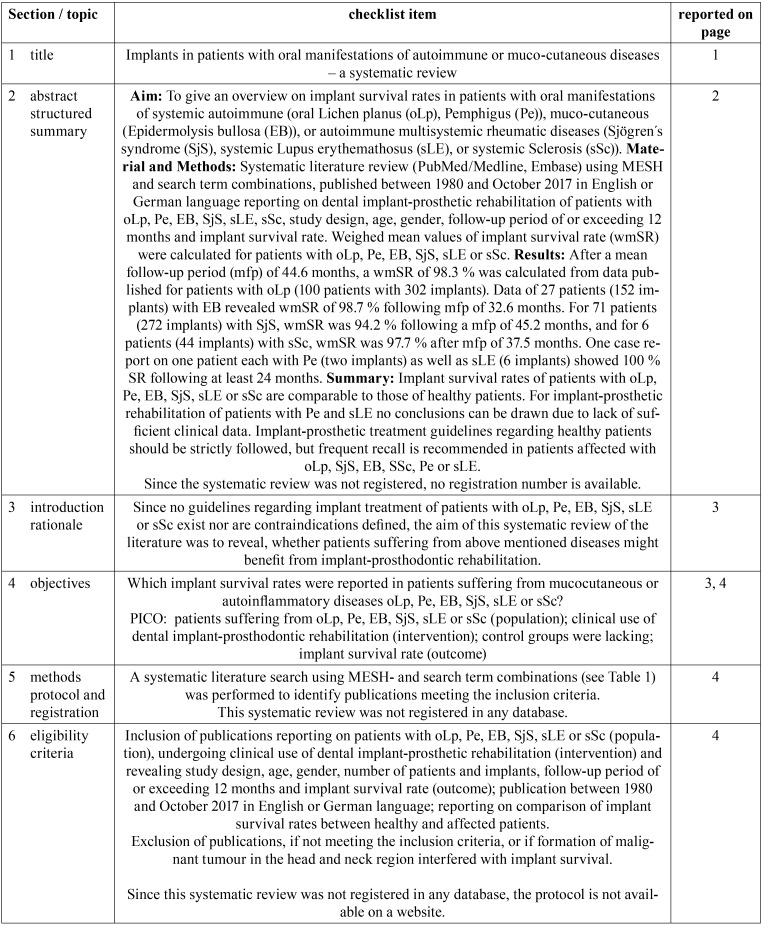
PRISMA checklist.

**Table 2:**
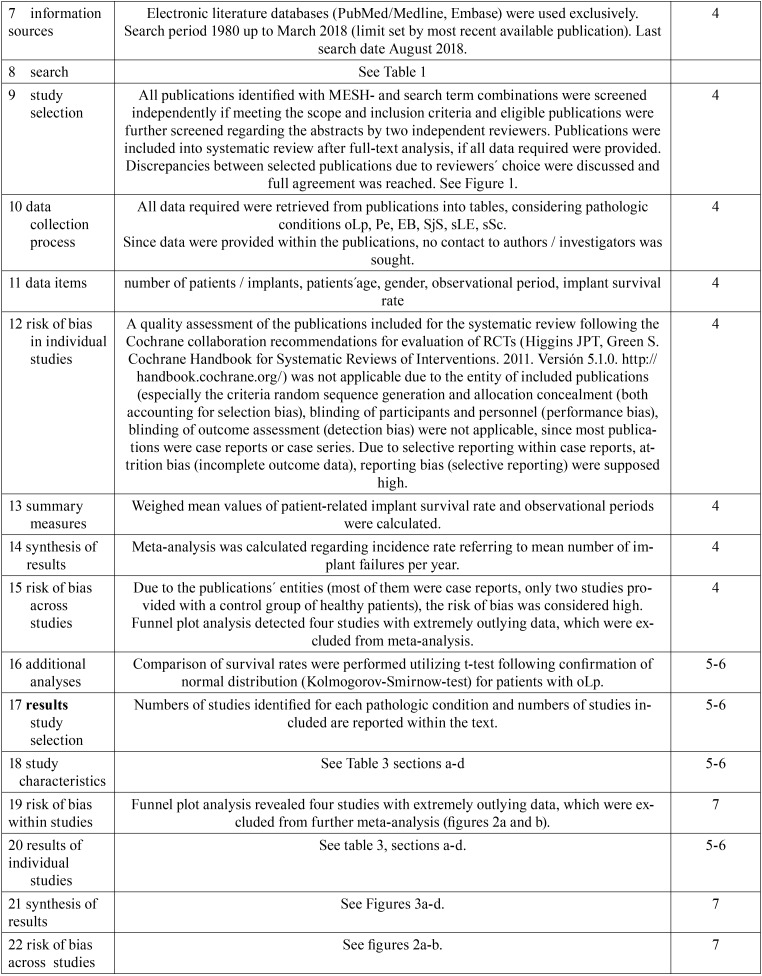
PRISMA checklist.

**Table 2:**
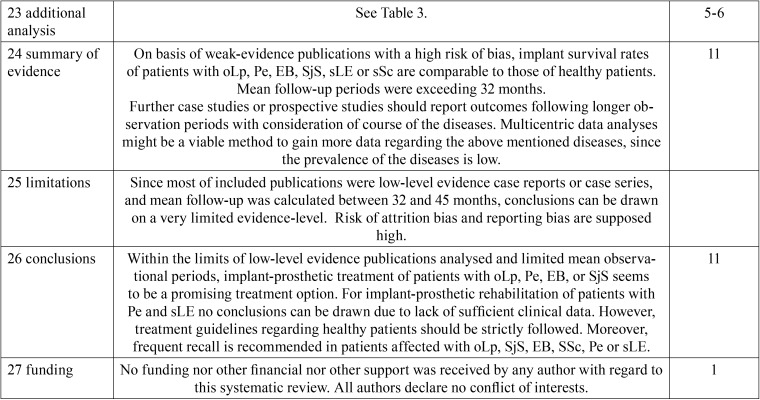
PRISMA checklist.

## Results

**Figure 1:**
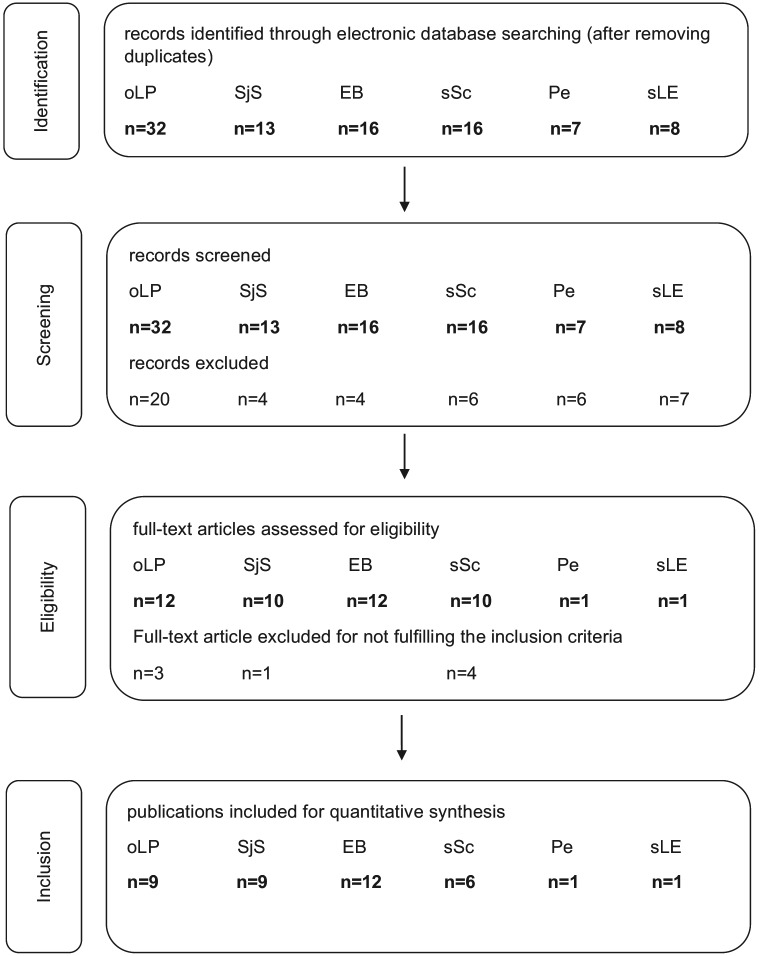
Search results, identification, screening for eligibility and inclusion of publications considered for systematic review and meta-analysis.

Figure 1 displays search results, identification, screening for eligibility and inclusion of publications considered for systematic review and meta-analysis. Comparison of inclusion of publications considering abstract analysis revealed a kappa-value of 0.932 (*p* < 0.0001). Disagreement regarding inclusion of two and exclusion of one publications was resolved after full text analysis, finally reaching full agreement.

The risk of bias assessment of included publications revealed an overall high risk of attrition bias and reporting bias, since case reports and case studies were included mainly. Assessment of random sequence generation, allocation concealment, blinding of participants as well as outcome assessment (accounting for selection bias, performance bias and detection bias) was not applicable.

Data were retrieved from included publications and information on reference, study type, number of implants and patients and their age, duration of follow-up period as well as implant survival rates are listed in  Table 3,  Table 3 continue sections a to d.

**Table 3:**
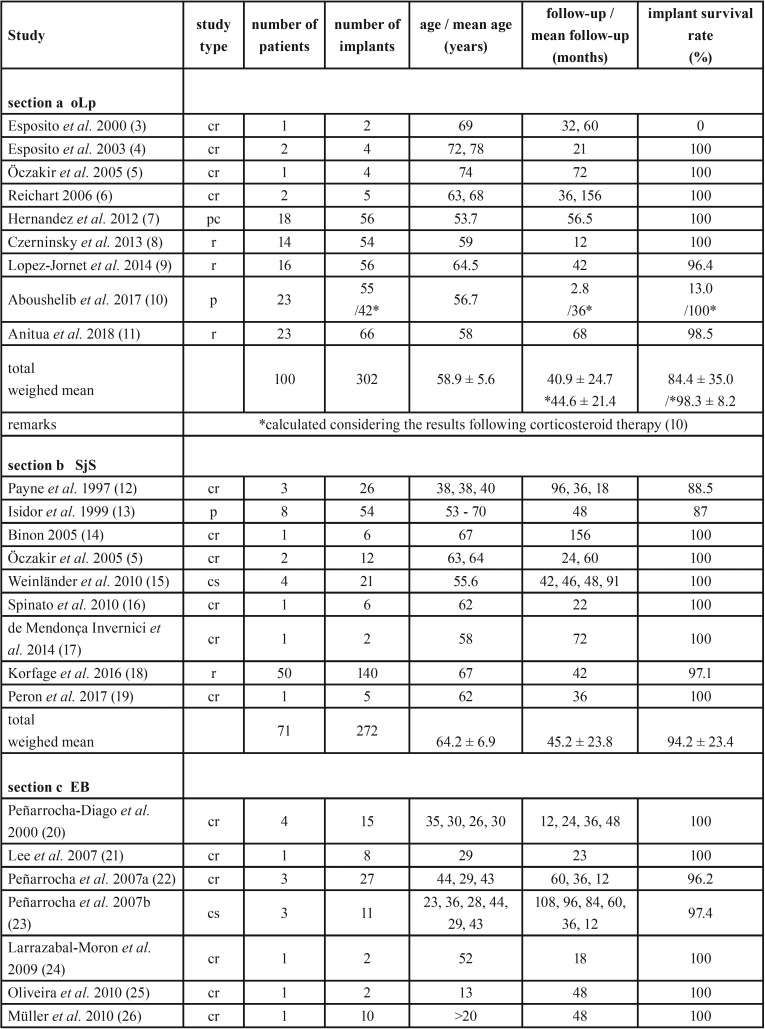
Study type, demographic data, duration of observation periods and implant survival rate of patients with oLp (section a), SjS (section b), EB (section c), sSc (section d).

**Table 3:**
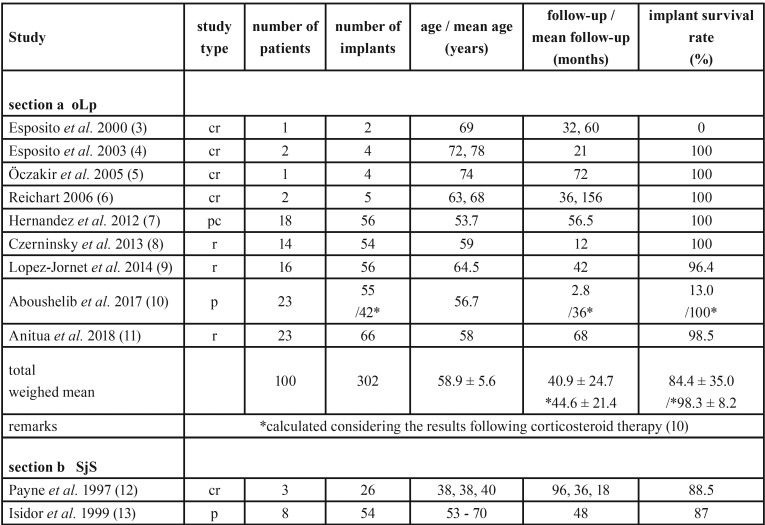
Study type, demographic data, duration of observation periods and implant survival rate of patients with oLp (section a), SjS (section b), EB (section c), sSc (section d).

-Oral Lichen planus

Nine out of 32 publications identified within databases met the inclusion criteria (four case reports, three retrospective studies, one prospective study, and one prospective controlled study). Data of 73 female and 27 male patients suffering from oLp were retrieved from these nine publications (see Table 3 section a). From the case report by Reichart (6) one patient was discarded due to unknown duration of follow-up period.

Despite lack of details regarding prosthodontic treatment for patient- or implant-based analysis in some publications (information on 38 % of patients as well as 37.8 % of implants were not available), the majority of patients (90.3 %) and implants (91.6 %) respectively were treated with fixed partial dentures, whereas only a few removable complete prostheses (1.6 % by patient-related and 2.1 % by implant-related analysis) and fixed complete prostheses (8.1 % by patient-related and 6.3 % by implant-related analysis) were used.

In one study, 55 implants were inserted during acute flare of active oLp using immediate implantation into the socket subsequently following extraction (17 implants) or immediate loading mode (31 implants), or both conditions (9 implants). 42 implants failed (24 immediately loaded, 6 immediately inserted) and were removed and replaced after oral corticosteroid therapy. None of the replaced implants failed (10).

In the study by Anitua *et al.* (11), short implants up to 8.5 mm in length were used exclusively in 8 patients with erosive and 15 patients with reticular oLp.

-Sjögren´s Syndrome

Nine publications of 13 identified within the databases met the inclusion criteria for the systematic review and revealed data of 65 female as well as 6 male patients suffering from SjS, receiving implant-prosthetic rehabilitation (six case reports and one case series, retrospective and prospective study, each, see  Table 3 section b). Data regarding distribution of type of prosthesis were available related to jaw (maxilla and or mandible) only. 92 jaws were treated. Of these, 44.6 % were treated using implant-retained removable complete dentures (overdentures). While counting implant-fixed single crowns (29.3 %) and fixed partial dentures (7.6 %) for fixed partial restorations (36.9 %) and counting 18.5 % fixed complete dentures, more jaws were treated using fixed restorations on implants (55.4 %) compared to removable prostheses.

-Epidermolysis bullosa

Data from 12 publications regarding implant-prosthetic rehabilitation of 27 patients (10 male and 17 female patients) with EB, receiving 152 implants were analysed (see  Table 3,  Table 3 continue section c). From the case series by Peñarrocha *et al.* (23) three patients with 27 implants were discarded since these were also subject of another publication (22).

Analysis by jaw (n = 41) revealed, that the majority of jaws were treated with implant-fixed complete dentures (70.7 %), whereas 22 % of jaws were treated with implant-retained removable complete dentures and 7.3 % with implant-fixed partial dentures.

-Systemic Sclerosis

Six publications out of 16 matching the search term combinations met the inclusion criteria, reported on treatment courses of one male and five female patients with sSc undergoing implant-prosthodontic rehabilitation (data listed in  Table 3,  Table 3 continue section d). Analysis per jaw revealed the use of four implant-fixed complete dentures, two implant-borne bar-retained removable complete dentures and three implant-fixed partial dentures (two were integrated in one maxilla).

-Pemphigus and systemic Lupus erythematosus

Of seven publications on Pe and eight publications on sLE identified, one publication was included each, reporting on one female patient (70 years old) suffering from Pe (2 implants, ball-attachment-retained mandibular overdenture, 32 months observational period) (36) and one female patient with sLE (49 years old, 6 implants, single fixed crowns, 24 months observational period) (37), both with 100 % implant survival.

-Meta-analyses

Meta-analysis was carried out, calculating a funnel plot to detect publication bias.

**Figure 2:**
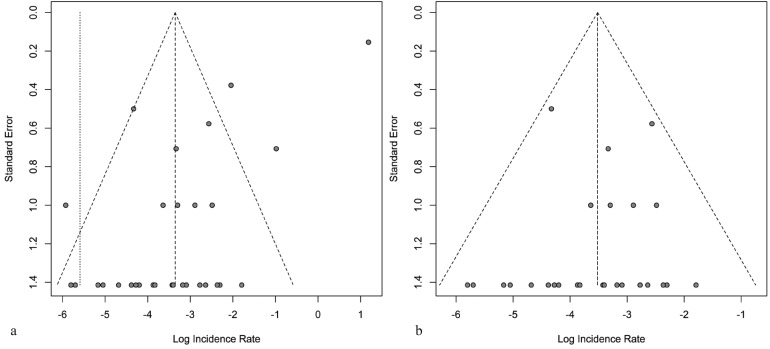
a) Funnel plot for all publications. Four publications (3,10 [including results prior to corticosteroid therapy in “active” oLP only],11,13) were found to reveal too extreme rate estimates for implant failure. b) Funnel plot for all publications following exclusion of outliers (3,10 [including results prior to corticosteroid therapy in “active” oLP only],11,13), revealing no risk of bias.

The funnel plot for all publications indicated that four publications (3,10 [including results prior to corticosteroid therapy in “active” oLP only],11,13) had to be excluded because their rate estimates for implant failure were too extreme (outliers) (see Figure 2a). Figure 2b shows the funnel plot, showing no asymmetry, revealing no evidence for further bias after removing the four publications as indicated. Thus, the remaining publications were included into further meta-analysis.

**Figure 3:**
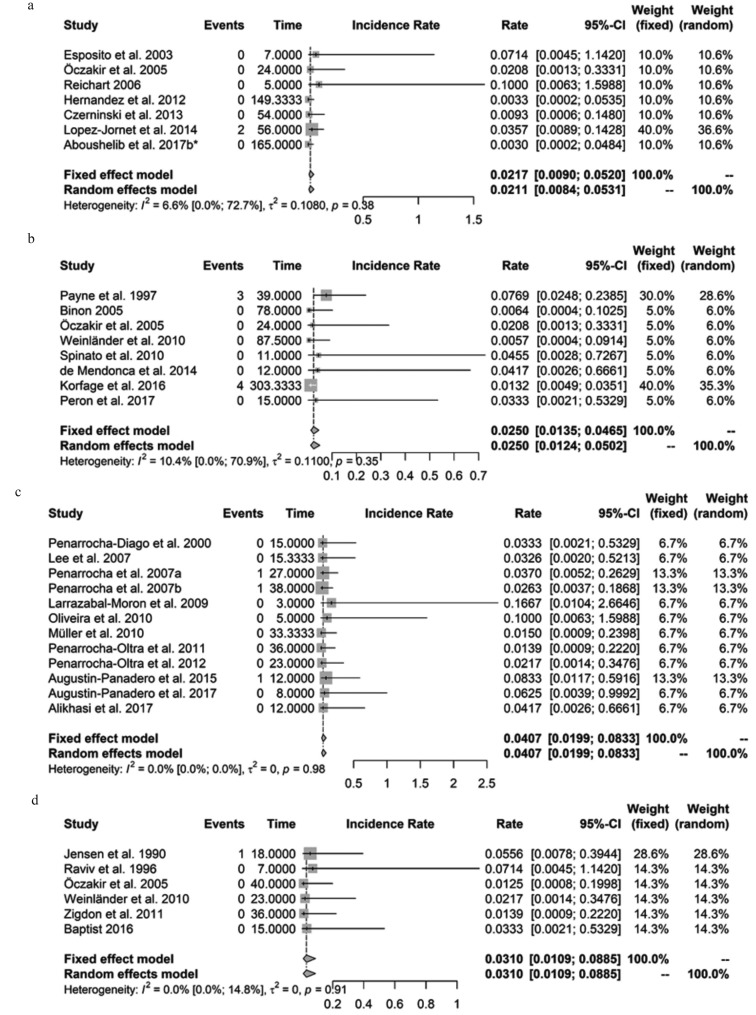
a) Forest plot for 7 publications on oLp. Incidence rate of mean number of implant failures per year: 0.0217 (CI95%: 0.0090; 0.0520, fixed effect model), no significant heterogeneity: I2=6.6% (CI95%: 0.0%; 72.7%); *p*=0.38.b) Forest plot for 8 publications on SjS. Incidence rate of mean number of implant failures per year: 0.0250 (CI95%: 0.0135; 0.0465, fixed effect model), no significant heterogeneity: I2=10.4% (CI95%: 0.0%; 70.9%); *p*=0.35. c) Forest plot for 12 publications on EB. Incidence rate of mean number of implant failures per year: 0.0407 (CI95%: 0.0199; 0.0833, fixed effect model), no significant heterogeneity: I2=0.0% (CI95%: 0.0%; 0.0%); *p*=0.98. d) Forest plot for 6 publications on sSc. Incidence rate of mean number of implant failures per year: 0.0310 (CI95%: 0.0109; 0.0885, fixed effect model), no significant heterogeneity: I2=0.0% (CI95%: 0.0%; 14.8%); *p*=0.91.

Figure 3 shows the forest plot for all included publications (n=35) on oLp, SjS, EB, sSc, Pe, and sLe revealing a considerably low incidence rate referring to mean number of implant failures per year of 0.0297 (fixed effect model). Figures 3a-d show forest plots for data, retrieved from publications on oLp, SjS, EB, and sSc, exclusively. Furthermore, measures for heterogeneity I2 for all included studies and for the subgroups regarding oLp, SjS, EB, sSc, Pe, and sLe were calculated. In all instances I2 was small and not significantly different from zero.

## Discussion

Gingiva and other oral tissues may exhibit several pathologic phenomena as symptoms or manifestations of systemic diseases, requiring further diagnostic and interdisciplinary treatment (38). Although epidemiological data indicate low prevalence with regional variations for oLp (1 – 2 % (39)), SjS (0.5 – 1 % (40)), and sLe (0.05 % (41)), or rare prevalence for EB (up to 0.001 % (42)), sSc (up to 0.03 % (43)), and Pe (0.05 % (44)), these patients affected require dental treatment, including prosthetic rehabilitation. Oral conditions in auto-inflammatory, autoimmune, rheumatic or muco-cutaneous diseases require special care due to oral lesions (ulcer- or blister formation, enhanced risk for malignant tumours), sicca symptoms, scar tissue formation, limited mouth opening, and due to side effects of anti-inflammatory, immune-modulating medication. Consequently, quality of life of these patients is reduced. Burden related to disease as well as adverse effects of medications is high for the patients, challenging the dental professional due to limited treatment options regarding conventional prosthodontic therapy. Especially wearing mucosal-borne removable partial or complete prostheses is impeded if not impossible.

Decision-making for dental implant-retained or fixed prosthodontic devices in order to improve speaking, swallowing, chewing, quality of life (45) should consider a critical weighing of advantages and risks, comorbidities as well as disease-related medication adverse effects, possibly interacting with osseointegration and implant prognosis. Generally, in patients affected by conditions and diseases mentioned above, large-area contact between oral mucosa and artificial surface of prosthodontic device and retention of the total or partial denture, or reduction of contact area between prostheses and mucosa is pursued.

The numbers of publications regarding implant-prosthodontic treatment under the above mentioned conditions and pathoses are low in general. The evidence-level of 38 publications included into this systematic review was limited (among them 26 case reports, four case series, four retrospective studies, two prospective studies, one prospective controlled study). Therefore, decision making prior to any intervention is based on low level of external evidence, but should include knowledge, experience and internal evidence of the dentist as well as thorough analysis of any conditions of the individual patient to evaluate risks and expected benefit and to provide a thorough patient information. Interdisciplinary consultations are required. Status of disease progress and overall prognosis should be considered additionally.

-Oral Lichen planus

oLp is a chronic autoimmune and inflammatory disease and may manifest as asymptomatic or symptomatic. About 1/3 of affected patients reveal gingival manifestations. Patients complain about mucosal burning sensations or pain caused by epithelial desquamation, erosions and ulcerations. Therefore, any contact or friction between dentures and mucosa should be avoided or reduced.

Data analysis of 100 patients included into this systematic review showed, that implant-fixed partial as well as complete prostheses were utilized mainly for prosthodontic rehabilitation of patients suffering from oLp. Implant-retained removable complete dentures were used in only 1.6 % of patients. Considering those patients with active oLp and replacement of failed implants following systemic corticosteroid therapy in one study (10), weighed mean implant survival rate was calculated 98 % after a mean follow-up period of 44.6 months, which is comparable to data from healthy patients.

Implants in patients with erosive oLp revealed no significant reduction of survival in some publications (4,6-9,11), whereas Aboushelib *et al.* (10) reported a significant effect of “active” oLp on implant loss, especially for those immediately loaded. Following oral corticosteroid and local soft laser therapy and recovery, implant replacement was performed and no further implant failures were observed. Therefore, to prevent disturbance of wound healing or osseointegration it is recommended to omit any surgical intervention during active, erosive phases of oLp (3,6-8,10). Additionally, local treatment of erosions and ulcerations with clobetasoldipropionate is recommended (7). Strict patient adherence to regular and frequent follow-up appointments and oral hygiene instructions should be asserted not only to rule out inflammatory tissue response interfering with long-term survival of implants (peri-implant mucositis and peri-implantitis) (1), but also to early detect malignant transformation of oLp into OSCC.

-Sjögren’s Syndrome

Treatment courses of 71 patients suffering from SjS with a weighed mean age 64 years were published – female patients were treated predominantly. Within this systematic review, the weighed mean survival of implants of 94 % after a mean follow-up of 45 months was found slightly less regarding other autoimmune diseases considered within this systematic review.

SjS is a chronic systemic autoimmune disease. Due to affection of exocrine glands – salivary and lacrimal glands in particular – patients suffer from hyposalivation and xerophthalmia, resulting – among other burdens – in stomatitis sicca, xerostomia, burning sensations, and difficulties to swallow.

Prosthodontic rehabilitation – especially using mucosal-borne dentures – is impeded due to dryness of oral mucosa, causing mucosal sensations, pain, or ulcerations. However, within the cohort of published treatment courses included into this systematic review, nearly half of the jaws (44.6 %) were treated using implant-retained removable complete dentures, whereas 55.4 % of the jaws were treated with implant-fixed prosthodontic restorations.

Although biofilm accumulation with concomitant enhanced risk of peri-implant mucositis or peri-implantitis is suspected due to hyposalivation, relatively high mean weighed implant survival rate was found in patients with SjS.

Patients with secondary SjS and rheumatoid arthritis sometimes may reveal limited manual abilities to perform proper oral hygiene. This and possible necessity of anti-inflammatory as well as immunosuppressive medication possibly interfering not only with osseointegration but health of peri-implant tissue should be considered while risk-benefit assessment, in therapy planning and thorough patient information as well. Patients should follow regular and frequent recall to early detect peri-implant mucositis and indicators for oral hygiene deficit.

-Epidermolysis bullosa

EB as a rare, inherited, recessive disease of skin and mucosa, manifests by forming of trauma-induced bullae – often with subsequent scar formation – and pseudosyndactyly. Involvement of oral and gastrointestinal mucosa include recurrent bullae, scar formation, microstomia and ankyloglossum, shallow vestibular sulci, but also periodontitis, alveolar bone resorption with atrophy of the edentulous areas of maxilla and mandible – all interfering with conventional prosthodontic treatment - and enhanced predisposition for oral squamous cell carcinoma (46).

Data analyses of 27 patients (mean age 35 years) revealed a weighed mean implant survival rate of 98.7 % after a mean observation period of 33 months. More than 80 % of the jaws were treated with implant-fixed complete or partial dentures, whereas nearly 20 % of the jaws were treated with implant-retained removable overdentures with short dental arch rehabilitation to prevent impeded oral hygiene access due to limited posterior space and mouth opening.

-Systemic Sclerosis

SSc is an autoimmune multisystem rheumatic disease affecting connective tissue, and an inflammatory, vascular and sclerotic disease of the skin as well as of organs (lung, heart, gastrointestinal tract). Oral and facial clinical findings are mask-like face, thin vermilion border, radial perioral furrows, microstomia, sclerosis of the tongue-tie and induration of the tongue. Hyposalivation, microstomia, ankyloglossia, limited mouth opening but also minor manual skills interfere with oral hygiene ability. While providing with implant-fixed prosthodontic superstructures, reconstructing shortened dental arches, disadvantages of lack of removal of overdentures due to progression of losing manual dexterity and microstomia might be taken into account (47,48). Close cooperation between dental professionals and rheumatologists is necessary for decision making, in treatment planning and maintenance.

Data on dental implant treatment courses of six patients suffering from sSc were available from 6 case reports, revealing a weighed mean implant survival rate of 97.7 % after a mean observation period of 37.5 months. Implant-fixed complete or partial dentures were used mainly and two patients received implant-retained removable overdentures.

-Pemphigus and systemic Lupus Erythematosus

Pe is an autoimmune mucocutaneous disease, characterized by epithelial blistering at skin and mucosa, leaving erosions or ulcers following rupturing affecting not only the oral cavity but also the mucosa of nose, conjunctivae, genitals, esophagus, pharynx and larynx.

SLE is a multisystem autoimmune disease with connective tissue and blood vessel disorder, characterized by episodes of recurrent acute or chronic inflammation with intermediate phases of remission, mainly affecting joints, internal organs and skin. Oral manifestations are common – especially during disease flares – and present as forms of painless ulcers (49). Treatment of both Pe and sLE comprises immunosuppressant medications mainly, which may cause adverse effects (among them candida-infections of the oral mucosa) with individually different degree of affection, and dependent on dosage and duration of use. As usual in dental treatment of special needs patients or patients with systemic autoimmune or mucocutaneous diseases, close cooperation of the dentist and the treating specialists is mandatory.

Limited mouth opening and hyposalivation due to secondary Sjögren’s syndrome as associated co-morbidity (50) can interfere with wearing of mucosal-borne dentures. Since ill-fitting prostheses or any traumatic contact with the mucosa can cause formation of vesiculobullous or ulcerative lesions, implant-borne stabilization of dentures will result in less trauma and higher patient comfort (36,37).

Only two case reports were found with data on one female patient each with Pe and sLE, both revealing 100 % implant survival after a follow-up period of at least 24 months, which is too less data to offer any recommendations. However, patients with Pe or sLE seem to benefit from implant-retained or implant-fixed prosthodontic treatment.

## Conclusions

Guidelines regarding implant treatment of patients with oLp, Pe, EB, SjS, sLE or sSc do not exist so far. Results of mainly low-evidence publications demonstrate encouraging outcomes regarding dental implant survival, which are comparable to those of healthy patients. Patients with the above mentioned diseases seem to benefit from implant-retained or implant-fixed prostheses. Disease-related contraindicating conditions cannot be defined.

However, for implant-prosthetic rehabilitation of patients with Pe and sLE no conclusions can be drawn due to lack of sufficient clinical data.

Implant-prosthetic treatment guidelines regarding healthy patients should be strictly followed, close recall intervals and full therapy adherence by the patients is required, and close cooperation of the dental professional and the treating specialists is mandatory in patients affected with oLp, Pe, EB, SjS, sLE or sSc.
